# Skin Grafting in the Colored Races

**Published:** 1878-10

**Authors:** 


Skin Grafting in the Colored Races.—A French naval surgeon, Dr. Maurel, stated at a scientific meeting in Paris, that during a two years' residence in Guiana, he had made numerous experiments on epidermic transplantation, placing the graft on persons of different race and color. He found that not only did the graft take well, whatever description of transplantation was made—whether transported from the skin of a black to that of a white, or the reverse—but that there always remained a whitish line at the point of junction, wherein pigmentation was not produced. The pigment disappeared from a black to a white person; but when the two individuals were highly colored, the graft remained black, except at the point of the cicatrization.—*Med. & Surg. Rep.*, August 10, 1878.

